# Respiratory symptoms and associated factors among workers in the marble factory in ethiopia: A comparative cross-sectional study

**DOI:** 10.1038/s41598-025-93323-8

**Published:** 2025-03-26

**Authors:** Ephrem Bogale, Belayneh Jabur, Chala Daba, Zemachu Ashuro, Samson Wakuma Abaya

**Affiliations:** 1 Department of Community Health Insurance Arada Sub-city Health Office, Addis Ababa, Ethiopia; 2 Department of Infection Prevention and Control Ministry of Health Saint Peter Specialized Hospital, Addis Ababa, Ethiopia; 3 Department of Environmental Health, College of Medicine and Health Sciences, Wollo University, Dessie, Ethiopia; 4 School of Public Health, College of Health Science and Medicine, Dilla University, Dilla, Ethiopia; 5 Department of Preventive Medicine, School of Public Health, College of Health Sciences, Addis Ababa University, Addis Ababa, Ethiopia

**Keywords:** Respiratory symptoms, Marble dust, Workers, Ethiopia, Diseases, Health occupations, Risk factors, Signs and symptoms

## Abstract

Occupational dust exposure is one of the major risk factors for respiratory health in many dust-generating work environments, including the marble industries. The objective of this study was to determine the prevalence of respiratory symptoms and associated factors among marble factories workers in Addis Ababa, Ethiopia, compared to non-dust exposed alcohol factory workers. A comparative cross-sectional study was conducted among randomly selected 246 marble factories workers and 246 alcohol factory workers. The respiratory symptoms were assessed by using the standardized questionnaires adopted from both British Medical Research Council (BMRC) Questionnaires and American Thoracic Society (ATS) questionnaires. The data were entered into Epi-data version 3.1 and analyzed using SPSS version 23. A Poisson regression analysis was performed to compare the prevalence of respiratory symptoms among marble factory workers and alcohol factory workers. A multivariable logistic regression analysis was done to identify factors that were significantly associated with respiratory symptoms. Statistical significance was determined at a p-value of less than 0.05. A Poisson regression analysis revealed that the prevalence of chronic respiratory symptoms was significantly higher among marble factory workers (42.1%) than among control workers (16.2%). Chronic Respiratory symptoms was significantly associated with age ≥ 40 years (Adjusted Odds Ratio [AOR] = 1.72, 95% CI:1.07–2.74), past history of respiratory illnesses (AOR = 5.07, 95% CI:3.23–7.96), not using respiratory protective Equipment (AOR = 2.16, 95% CI: 1.19–3.92), work experience ≥ 10 years (AOR = 2.04, 95% CI:1.12–3.70), and working hours ≥ 48 h per week (AOR = 2.19, 95%CI: 1.36–3.56). Thus, respiratory protection equipment and engineering control methods should be implemented to reduce workers’ exposure to marble dust in marble factories.

## Introduction

Occupational dust exposure leads to a high prevalence of lung function impairment^1^. Marble workers are continually exposed to dust particles, which include calcium carbonate and silica. Long-term exposure to respirable crystalline silica can lead to silicosis. Besides from silicosis, respirable crystalline silica has been associated to autoimmune illnesses, non-malignant renal disease, chronic obstructive pulmonary diseases (COPD), and lung cancer^2^.

In the marble industry, there are cutting, polishing, and finishing sections that generate a large amount of marble dust^3^. During the marble cutting process, around one-third of the initial mass of marble is lost in the form of dust that is suspended in the air and subsequently inhaled by the workers^4,5^.

Non-communicable diseases are responsible for an estimated 82% of all deaths worldwide. Chronic respiratory illnesses, asthma, and COPD accounted for 4 million, or 10.7% of deaths^6^. Chronic respiratory diseases pose a public health problem in both industrialized and developing countries because of their health and economic implications^4^.

A study found that chronic respiratory symptoms such as chronic cough, chronic phlegm, wheezing, shortness of breath, and chest pain are manifestations of respiratory problems that are mostly caused by occupational exposures^7^. In addition, an epidemiological study indicated that workers exposed to marble dust have an increased risk of suffering from chronic disease^5^. Besides, lung irritation, lung function impairment, lung inflammation, chronic obstructive lung disease, restrictive lung disease, and pneumoconiosis are caused by dust particles that are inhaled and lodged in the lung^8^. The study also found that cough, shortness of breath, chest pain, chest tightness, and abnormal breathing patterns are chronic respiratory symptoms continually suffered by marble industry workers in developed as well as developing countries^9^.

Many studies have been conducted in Ethiopia on the effects of organic dust on respiratory health, but there is a lack of research on inorganic dust effects. No study has been conducted on the respiratory symptoms and associated factors among marble factory workers. This is a clear gap in the area; hence the objective of this study was to determine the magnitude of respiratory symptoms and associated factors among marble factory workers in Addis Ababa, Ethiopia.

## Materials and methods

### Study design, setting and period

There are about 12 marble factories in Addis Ababa, Ethiopia. Considering the available resources, we selected three marble factories and one alcohol factory for comparison. From January to March 2021, a comparative cross-sectional study was conducted among workers in three selected marble factories as well as control workers in one alcohol factory. Marble production is rising in the city especially during the previous decades, a number of marble deposits throughout the country have been put into production in factories located in the capital city Addis Ababa.

### Sample size determination

The sample size for this study was determined using a double population proportion formula, with the following assumptions: a 95% confidence interval (CI), 80% power. We determined sample size by taking previous study findings on respiratory symptoms and ventilator function among quarry workers in Edo state, Nigeria, which found that the prevalence of cough was 23.7 and 13.5% in the exposed and non-exposed groups, respectively^10^.

$$\:n\:=\frac{{\left(Z\alpha\:/2+Z\beta\:\right)}^{2}*(P1\left(1-P1\right)+P2\left(1-p2\right))}{(P1-P2{)}^{2}}$$

Where,

n = the required number of sample size.

P1 = proportion of cough among the exposed group from previous study.

P2 = proportion of cough among the control group.

Z(α/2) = 95%confidence level statistical value (1.96).

Zβ = the desired power of 80% = 0.84.

$$\:n\:=\frac{{(1.96+0.84)}^{2}*(0.237(1-0.237)+0.135(1-0.135)}{(0.237-0.135)^2} = 224$$

Adding 10% for non-response rate, the final sample size for each group became 246 with a total sample size of 492.

## Source and study population

The source population for this study was all workers employed in the three selected marble factories and one alcohol factory in Ethiopia. The study population was workers engaged in the production process in marble and alcohol factories.

### Eligibility criteria

In this study, we included workers directly engaged in production activities in marble factories who had worked there for at least one year and were over the age of 18 years as the marble dust exposed group, while workers from the alcohol factory were chosen as the control group due to their low dust exposure status. Workers with acute illnesses were excluded from the study.

### Sampling procedure

First, we stratified the work process in both marble and alcohol factories by production unit, assuming that workers in different production units would be exposed to different levels of dust. The sample was subsequently determined using systematic sampling techniques, and the workers’ roster was used as a sample frame. The first study participant was randomly selected from each production unit, and the other study participants were selected every fourth interval from the workers’ rosters.

### Data collection tools and procedures

Data was collected using both British Medical Research Council (BMRC) respiratory questionnaires and the American Thoracic Society (ATS) Questionnaires after performing some modifications^11^. The questionnaire was translated from English to Amharic and then back to English to maintain its consistency. To assure data quality, we performed a pre-test on the adopted questionnaires before data collecting. Before administering the questionnaire by face-to-face interview by health professionals, we gave the study participants a brief explanation of the purpose of the study in order to obtain informed consent. A workplace observation checklist was employed to assess the use of personal protective equipment, housing condtion, type of machine used, and the ventilation status of the workplace (natural ventilation: window to floor area ratio was measured, while artificial ventilation availability and functionality were assessed).

### Measurement of variables

***Respiratory symptom***: defined as the development of one or more symptoms of cough, phlegm, shortness of breath, wheezing, and chest tightness that last at least three months in one year ^12,13^.

***Cough*** is defined as the development of coughing as much as 4–6 times a day on most days of the week for at least three months in one year^12,13^.

***Phlegm*** is defined as sputum expectoration of up to twice per day for most days of the week for at least three months in a year^12,13^.

***Wheezing*** is defined as the state of causing a wheezing or whistling sound during inspiration or expiration for at least three months in a year^12,13^.

***Chest tightness***: is sensation of chest congestion or constriction or difficulty with deep inspiration while at work or just after work^12,13^.

***Shortness of breath*** is defined as a discomfort or difficulty breathing in different activities, such as walking up a slight hill, when undressing, or walking at your own pace^12,13^.

Past dust exposure any work experience on dusty environment before the current working position^12,13^.

***A family history of chronic respiratory disease*** is defined as the presence of one or more chronic diseases such as chronic bronchitis, emphysema, tuberculosis (TB), chronic sinus, asthma, and lung cancer in either of the natural parents (mother or father) documented by physicians^12,13^.

***Past respiratory disease*** is defined as one or more of respiratory diseases such chronic bronchitis, emphysema, tuberculosis (TB), chronic sinus, asthma, and lung cancer that could have occurred before the current working position and were diagnosed by physicians^12,13^.

A **current smoker** is defined as a worker who smoked at the time of the study or had stopped smoking less than one year before, an **ex-smoker** is a worker who had quit at least one year before the study, and an ***ever smoker*** is a worker who has smoked at least one hundred cigarettes in his or her life, which includes current smokers and ex-smokers^12,13^.

### Statistical analysis

The data was entered into Epi-data version 3.1 and then exported to SPSS version 23 for analysis. The data was cleaned to verify that it was complete and consistent. Descriptive statistics were used to summarize the results. The Pearson chi-square test was used to determine the difference between the groups for the categorical variables. A Poisson regression analysis with a robust variance was used to determine the prevalence ratio of respiratory symptoms (cough, phlegm, wheezing, shortness of breath, and chest pain) between marble factory workers and alcohol factory workers, after adjusting for education level, age, monthly income, previous respiratory disease, service year, working hours per week, and safety training. Variables with *p* < 0.2 in the bivariable analysis were included in the multivariable analysis. To find statistically significant associations in the final model, we used adjusted odds ratios (AOR), 95% confidence intervals (CI), and *p* < 0.05. Multi-collinearity was tested using the variance inflation factor (VIF), and model fitness was assessed using the Hosmer and Lemeshow goodness-of-fit test at p-values greater than 0.05.

### Ethical approval and consent to participate

This study was approved by the institutional review board of Addis Ababa University College of Health Science School of Public Health. The data was collected after obtaining permission from factory managers following the submission of a supportive letter and ethical approval letter from Addis Ababa University. Before beginning data collection, we obtained written informed consent from each study participant by explaining the objective, benefits, and risks of participating in this study. All methods were performed according to the relevant guidelines and regulations.

## Results

### Socio-demographic characteristics of study participants

In this study, 242 marble workers and 229 controls participated, with a 95.7% response rate. The reasons for non-response were that seven workers refused to participate, six workers were on sick leave, five workers were on annually leave, and three workers had left employment. The majority of the study’s participants were male, married, and permanently employed. The marble workers had earned low income (< 3000 ETB) compared to the controls. However, there was no statistically significant difference between marble workers and controls in terms of sex, age, religion, educational status, and work experience (Table 1***)***.

**Table 1:** Socio-demographic characteristics of study participants in the marble factories and among controls in addis Ababa, Ethiopia, 2021 (*n* = 471).

Variables	Category	Marble workers (*n* = 242)	Controls (*n* = 229)	Total	p-value
		n (%)	n (%)	n (%)	
Sex	Male	194 (80.2)	169 (73.8)	363 (77.1)	0.101 ^a^
	Female	48 (19.8)	60 (26.2)	108 (22.9)	
Age	≤ 29 years	35 (14.5)	37 (16.2)	72 (15.3)	0.058 ^a^
	30–39 years	71 (29.3)	68 (29.7)	139 (29.5)	
	≥ 40 years	136 (56.2)	124 (54.1)	260 (55.2)	
Religion	Orthodox	184 (76)	164 (71.6)	348 (73.9)	0.094 ^a^
	Muslim	34 (14)	34 (14.8)	68 (14.4)	
	Protestant	24 (10)	31 (13.5)	55 (11.7)	
Marital status	Married	158 (65.3)	151 (65.9)	309 (65.6)	0.035 ^a^
	Single	64 (26.4)	59 (25.8)	123 (26.1)	
	Divorced	16 (6.6)	15 (6.6)	31 (6.6)	
	Widowed	4 (1.7)	4 (1.7)	8 (1.7)	
Educational status	Can’t read and write	6 (2.5)	6 (2.6)	12 (2.5)	0.055 ^a^
	Primary	83 (34.3)	76 (33.2)	159 (33.8)	
	Secondary	103 (42.5)	100 (43.7)	203 (43.1)	
	Diploma and above	50 (20.7)	47 (20.5)	97 (20.6)	
Employment condition	Temporary	18 (7.4)	17 (7.4)	35 (7.4)	0.005 ^a^
	Permanent	224 (92.6)	212 (92.6)	436 (92.6)	
Monthly income	< 3000 ETB	151 (62.4)	56 (24.5)	207 (43.9)	0.001^a^
	≥ 3000 ETB	91 (37.6)	173 (75.5)	264 (56.1)	
Work experience	≤ 4 years	45 (18.6)	39 (17)	84 (17.8)	0.882 ^a^
	5–9 years	51 (21.1)	53 (23.1)	104 (22.1)	
	> 10 years	146 (60.3)	137 (59.8)	283 (60.1)	

*“n”: number of study participants;* ^*a*^ *Pearson chi-square test; ETB: Ethiopian Birr; controls were employees of the alcohol factory*.

### Behavioural and work-related characterstics of the participants

In this study, marble industry workers had a higher past history of past history of respiratory illnesses and dust exposure than control workers. The majority of study participants, 357(76.7%), did not wear respiratory protective equipment in the workplace. In this study, there was no statistically significant difference between marble workers and controls concerning current smokers, ever smokers, family history of respiratory illnesses, biomass fuel use, workplace supervision, and health and safety training ***(*** Table 2***).***

**Table 2:** Behavioural and work-related characteristics of the study participants in the marble factories and among controls (*n* = 471).

Variables	Marble workers (*n* = 242)	Controls (*n* = 229)	Total	*p*-value
	*n* (%)	*n* (%)	*n* (%)	
Past history of respiratory illnesses	66 (27.3)	48 (21.0)	114 (24.2)	0.001 ^*a*^
Family history of respiratory illnesses	45 (18.6)	30 (13.1)	75 (15.9)	0.180 ^a^
Current smokers	10 (4.1)	9 (3.9)	19 (4)	0.078 ^a^
Ever smokers	28 (11.6)	26 (11.4)	54 (11.5)	0.277
Past dust exposure history	50 (20.7)	43 (18.8)	93 (19.7)	0.001 ^*a*^
Participants who use respiratory protective equipment	56 (23.1)	54 (23.6)	110 (23.3)	0.028 ^*a*^
Participants who had taken health and safety training	64 (26.4)	60 (26.2)	124 (26.3)	0.826 ^a^
Workplace supervision	78 (32.2)	76 (33.2)	154 (32.7)	0.101 ^a^
Use of biomass fuel for cooking	151 (62.4)	135 (58.9)	286 (60.7)	0.418 ^a^

*“n”: number of study participants;* ^*a*^ *pearson chi-square test; controls were employees of the alcohol factory*.

### Prevalence of chronic respiratory symptoms

The prevalence of chronic respiratory symptoms was higher among marble factory workers (17.8–30.6%) than the control workers (3.5–16.2%). After adjusting for age, educational level, monthly income, previous respiratory disease, work experiences, working hours per week, and health and safety training, marble factory workers had a statistically significant higher prevalence ratio of all chronic respiratory symptoms than controls. Furthermore, shortness of breath (30.6%) was the most prevalent chronic respiratory symptom reported among marble factory workers. Moreover, 42.1% of marble factory study participants and 16.2% of control workers reported having at least one respiratory symptom ***(***Table 3***).***

**Table 3:** Prevalence of chronic respiratory symptoms among marble factories workers and controls in addis Ababa Ethiopia, 2021 (*n* = 471).

Variable	Marble workers	Controls	Total	PR _adj_, 95% CI
	*n* (%)	*n* (%)	*n* (%)	
Cough	64 (26.4)	18 (7.9)	82 (17.4)	4.21 (2.41–7.38) *
Phlegm	56 (23.1)	11 (4.8)	67 (14.2)	5.97 (3.04–11.72) *
Wheezing	43 (17.8)	8 (3.5)	51 (10.8)	5.96 (2.74-13.00) *
Shortness of breath	74 (30.6)	15 (6.6)	89 (18.9)	6.28 (3.48–11.34) *
Chest tightness	70 (28.9)	13 (5.7)	83 (17.6)	6.76 (3.62–12.63) *
At least one respiratory symptom	102 (42.1)	37 (16.2)	139 (29.5)	3.78 (2.45–5.84) *

PR: Prevalence Ratio; “n”: number of study participants; *: p < 0.05; CI: Confidence Interval while adjusting for education level,* age*,* monthly income*,* previous respiratory disease*,* work experiences*,* working hours per week*,* and safety training; controls were employees of the alcohol factory*.

### Workplace observation findings

The results of workplace observations and area measurements revealed that there was inadequate natural and artificial ventilation. Dust accumulated on the walls, ceilings, floors, and in the production departments (cutting, polishing, and finishing). However, a wet method was used to reduce dust and cool the machine. The majority of the machines were old and lacked a dust sucker and remover. There was no regular supply of personal protective equipment, and those that were available during data collection were of poor quality.

### Factors associated with chronic respiratory symptoms

Variables with a p-value less than 0.2 in bivariable logistic regression analysis are eligible for multivariable logistic regression analysis, including age, monthly income, past history of respiratory illnesses, family history of respiratory disease, current smoker, respiratory protective equipment use, type of energy used at home for cooking, work experience, working hours per week, and workplace supervision. In a multivariable logistic regression analysis, age, past history of respiratory illnesses, usage of respiratory protective equipment, work experience, and working hours per week were significantly associated with chronic respiratory symptoms at *p* < 0.05.

In this study, study participants over 40 years of age were 1.72 times (AOR = 1.72, 95%CI: 1.07–2.74) as likely to have respiratory symptoms than those under 29 years of age. The odds of having chronic respiratory symptoms after 10 years of work experience was 2.04 times higher (AOR = 2.04, 95% CI 1.12–3.70) than workers who had work experience of less than or equal to 4 years. Workers with a history of respiratory illnesses were 5.07 times as likely to have respiratory symptoms (AOR = 5.07, 95%CI: 3.23–7.96) than those with no history of respiratory illnesses.

Workers who didn’t use respiratory protection equipment was 2.16 times (AOR = 2.16, 95%CI: 1.19–3.92) as likely to have respiratory symptoms than those who did. The number of working hours per week in the factory was also significantly associated to the chronic respiratory symptoms. The number of hours worked per week in the factory was also significantly associated with chronic respiratory symptoms. Workers who worked ≥ 48 h per week had 2.19 times (AOR = 2.19, 95%CI: 1.36–3.56) higher odds of experiencing chronic respiratory symptoms compared to those who worked less than 48 h **(**Table 4*).*

**Table 4:** Multivariate analysis result of factors associated with chronic respiratory symptoms among marble factory workers and controls in addis Ababa, Ethiopia, 2021 (*n* = 471).

Variables	Respiratory symptoms		COR (95%CI)	*p*-value	AOR (95%CI)	*p*-value
	Yes	No				
Age ≤ 29 years 30–39 years ≥ 40 years	20 33 86	52 106 174	1.00 1.23 (0.65–2.36) 0.78 (0.44–1.38)	0.143 0.394	1.00 0.63 (0.28–1.47) 1.72 (1.07–2.74)	0.285 0.025*
**Monthly income** ≥ 3000 ETB <3000 ETB	66 73	198 134	1.00 1.63 (1.09–2.43)	0.016	1.00 0.77 (0.47–1.28)	0.317
**Past history of respiratory illnesses** Yes No	65 74	49 283	5.07 (3.23–7.96) 1.00	0.001	5.07 (3.23–7.96) 1.00	0.001*
**Family history of respiratory illnesses** Yes No	38 101	37 295	3.0 (1.81–4.97) 1.00	0.001	0.62 (0.33–1.16) 1.00	0.136
**Current smoker** Yes No	1 138	18 314	7.91 (1.05–59.85) 1.00	0.045	4.94 (0.63–38.83) 1.00	0.129
**Uses of respiratory protective equipment** Yes No	38 101	72 260	1.00 0.74 (0.47–1.16)	0.187	1.00 2.16 (1.19–3.92)	0.025*
**Work experience** ≤ 4 years 5–9 years ≥ 10 years	34 31 74	50 73 209	1.00 0.52 (0.31–0.87) 0.834 (0.51–1.37)	0.012 0.473	1.00 1.77 (0.88–3.56) 2.04 (1.12–3.70)	0.109 0.020*
**Working hours per week** ≥ 48 h < 48 h	59 80	196 136	1.95 (1.31–2.92) 1.00	0.001	2.19 (1.36–3.56) 1.00	0.001*
**Workplace Supervision** Yes No	37 102	117 215	1.50 (0.97–2.33) 1.00	0.070	1.38 (0.76–2.52) 1.00	0.29
**Type of Energy used at home for cooking** Biomass fuel Electricity	93 46	193 139	1.46 (0.96–2.21) 1.00	0.076	0.98 (0.60–1.60) 1.00	0.935

*p-value < 0.05, AOR: Adjusted Odds Ratio, CI: Confidence Intervals, COR: Crude Odds Ratio, ETB: Ethiopian Birr.

## Discussion

In this study, the overall prevalence of chronic respiratory symptoms among marble factory workers was significantly higher than that of controls (42.1% vs. 16.2%), with chronic shortness of breath (30.6% vs. 6.6%), chronic chest tightness (28.9% vs. 5.7%), and chronic cough (26.4% vs.7.9%). Moreover, age, previous respiratory disease, use of personal protective equipment, service year, and weekly working hours were found as statistically significant factors associated with respiratory symptoms among marble factory workers.

Marble factory workers had a higher prevalence of respiratory symptoms than controls, but it was lower than the study conducted among quarry workers in Rio de Janeiro (65.3%)^14,15^.

Furthermore, when compared to controls, marble factory workers showed a higher prevalence of all respiratory symptoms, including cough, phlegm, wheezing, shortness of breath, and chest tightness. This respiratory complaint was observed in previous studies conducted among quarry workers in Ebonyi State, Nigeria^16^, and marble cutting workers in India^9^. This revealed that workers in marble factories are exposed to marble dust that contains silica, which is responsible for the occurrence of chronic respiratory symptoms.

Shortness of breath was the most common respiratory symptom reported in this study (30.6%). This study result was consistent with a study conducted in India, where the most common reported respiratory symptom was shortness of breath (26%)^9^. However, in studies carried out among quarry workers in Ebonyi State, Nigeria^16^ and in Iran^9^ among marble cutting workers, the most prevalent reported respiratory complaint was cough with a prevalence of 31.9% and 47.6%, respectively, while expectoration (41.7%)^15^ was the most common reported respiratory symptom in a study conducted in Rio de Janeiro among quarry workers. The possible explanations for the variations in the results could be technological use, sample size, work environment, and study change. Furthermore, the disparity could be explained by differences in the use of Personal Protective Equipment (PPE). Only 23.1% of marble factory workers used personal protective equipment (PPE) in this study.

The current study showed that age, past respiratory disease, Personal Protective Equipment use, service year, and weekly working hours were all statistically significant variables associated with respiratory symptoms among marble manufacturing workers. This study indicated that workers over the age of 40 were 1.72 times more likely to experience respiratory symptoms (AOR = 1.72, 95% CI:1.07–2.74) than those under the age of 29. The result is in line with the findings of a research conducted at the Dejen Cement Factory^17^. In addition, a South African study found that exposed elderly people had a significantly higher prevalence of chronic respiratory symptoms and disorders than those who had not been exposed^18^. The higher prevalence of respiratory symptoms among workers over the age of 40 in this study may be explained by aging, because aging causes changes in anatomical, physiological, and immunological functions^19^.

Workers with more than ten years of experience were more likely to have respiratory symptoms, according to this study (AOR = 2.03, 95% CI: 1.12–3.70). The current study’s findings were supported by a study conducted on marble workers in Besole Village, Indonesia, which indicated that workers suffering from respiratory problems were those who worked for more than ten years^20^. The finding is also consistent with a 40-year follow-up research on whetstone cutters, which found that more than half of the workers died from chronic diseases and concluded that length of service is one of the key factors determining disease progression^21^. This revealed that the occurrence of lung function impairment and respiratory symptoms may be related to the duration of employment, exposure to dust, as well as individual susceptibility and the amount of exposure or dose of dust entering the body.

The study also found that those exposed for more than 48 h per week were more likely to develop chronic respiratory problems than those exposed for less than 48 h (AOR = 2.12, 95% CI:1.35–3.56). This could be associated with long-term exposure to increased accumulation of dust in the respiratory system, which can decrease lung function and cause respiratory symptoms.

Workers with confirmed chronic respiratory disorders were more likely to have chronic respiratory symptoms in this study (AOR = 5.07, 95% CI: 3.23–7.96). This study result, in line with a study done in Dejen cement factory workers, also showed that workers who had previous chronic respiratory disorders experienced chronic respiratory symptoms more likely than workers who had no previous chronic respiratory diseases^12^. This could be because occupational exposure to dust can cause occupational asthma and chronic obstructive lung disease, which can exacerbate the occurrence of respiratory symptoms. The study found that compliance to PPE use is a preventive factor that leads in a decrease in respiratory illnesses^16^. Workers who have the habit of wearing masks will decrease inhalation exposure when breathing^20^. In this study, the use of PPE was found to be strongly associated with chronic respiratory problems. Workers who didn’t use PPE were 2.16 times more likely to have respiratory symptoms than those who did (AOR = 2.16, 95%CI: 1.19–3.92). In this study, the vast majority of workers (73%), didn’t use personal protection equipment. The study’s findings were comparable to a study conducted in Nigeria, which indicated that 98.3% of quarry workers did not wear PPEs^16^. The reason for this was that the majority of the workers did not use proper respirators, which predisposes to the occurrence of respiratory symptoms and reduced lung function. Therefore, the use of proper respirators should be encouraged, which can be accomplished through health education, awareness-raising, and appropriate workplace training programs.

### Limitations of the study

The study used a cross-sectional design; therefore, a cause-and-effect link cannot be determined. Because respiratory symptoms are not always simple to remember, the workers may have recall bias in the assessment. Furthermore, workers with respiratory health issues may have quit their jobs, thus a healthy worker impact cannot be completely ruled out.

## Conclusion

When compared to controls, marble factory workers exposed to marble dust had a significantly higher prevalence of chronic respiratory symptoms and age, previous respiratory disease, working experience, workeng hour per week were found to be independent predictors of respiratory symptoms. Therefore, Health education programs should prioritize among workers, including sharing information about the occupational hazards and diseases and proper use and maintenance of personal protective equipment and it is necessary to reduce dust exposure hours, assess health status for early diagnosis and treatment. Moreover, workers should be rotated to avoid prolonged exposure to dusty conditions and managers must implement prevention strategies such as the use of wet processes to limit dust generation, exhaust natural and artificial ventilation in the workplace. Additionally, a follow-up study should be conducted to see the cause-and-effect relationship between marble dust exposure and respiratory health of marble production workers.

### Acronyms and abbreviations

AAU, Addis Ababa University: AOR: Adjusted Odds Ratio: ATS, American Thoracic Society BMRC: British Medical Research Council; COPD: Chronic Obstructive Pulmonary Disease; COR: Crude Odds Ratio: PPE, Personal Protective Equipment: SPSS: Statistical Packages for Social Science; WHO: World Health Organization.

### Ethical approval and Concent

The study was conduct after obtaining an ethical clearance from the Institutional Review Board of the College of Health Sciences of Addis Ababa University. In addition, permission to conduct the study was also obtained from the factorymanagers. Before performing the study written informed consent was obtained from each participant, and participants were informed that they have full right to refuse and withdraw at any time in the study.

## Data Availability

The datasets generated during and/or analyzed during the current study are available from the corresponding author on reasonable request.
